# Micro-CT Evaluation of Spontaneous Apexification of an Immature Tooth following Trauma

**DOI:** 10.1155/2023/3779225

**Published:** 2023-07-05

**Authors:** Peter Cathro, Mike Smith, Jithendra Ratnayake, Geoffrey Heithersay

**Affiliations:** ^1^Faculty of Dentistry, The University of Otago, 310 Great King Street, Dunedin 9016, New Zealand; ^2^School of Dentistry, Faculty of Health Science, The University of Adelaide, Adelaide SA, Australia

## Abstract

This case reports on the micro-computerised tomography (CT) images of a periapical calcified dome following spontaneous apexification as a subsequence of trauma. An immature, maxillary central incisor was found to be non-vital one month following trauma. The tooth had minimal signs of structural damage; however, there was radiographic evidence of spontaneous apexification. The tooth suffered a second traumatic episode two years later and was decoronated to facilitate bone retention during osseous development. The patient presented with swelling and pain 36 months later, and the tooth root was extracted. The root was embedded in resin, and cross-sectional images were obtained using micro-CT. Analysis of the images provided insight into the structure of the calcified dome that formed following the first traumatic injury.

## 1. Introduction

Traumatic dental injuries to young permanent teeth have become an important public health problem affecting 30% of children worldwide [1]. Dental injuries in the permanent dentition occur most commonly within the 8- to 12-year age groups, in which the erupting teeth exhibit short and incompletely formed roots surrounded by loosely structured periodontal ligament [1]. The majority of these traumatic incidents occur before root formation is complete and may result in pulpal inflammation or necrosis [2].

Traumatic injuries are classified by the degree of damage to the dental and supporting tissues [3] with crown fractures being the most commonly reported dental trauma in the permanent dentition [4]. Injuries that result only in enamel fractures are unlikely to have further complications and are most likely to have good long-term pulp survival [5]. However, if there is an intrusive injury then pulp canal obliteration or pulp necrosis can occur, which can pose potential problems, such as arrested root development [6].

Hertwig's epithelial root sheath (HERS) is responsible for the development of the root [7]. HERS is sensitive to trauma but root formation can sometimes continue even in the presence of necrosis or pulpal inflammation due to the degree of vascularity and cellularity in the apical region where the sheath acts as a reservoir for undifferentiated cells that can differentiate into cells that form hard tissue [2, 8, 9]. Destruction of HERS results in cessation of normal root development leaving an immature tooth with a wide-open apex and thin root canal walls [9]. Therefore, it is important to make every effort to maintain the viability of HERS since it plays an important role in continuous root development after pulpal injury [10].

Treatment options for traumatised immature teeth with pulp damage include pulpotomy, apexogenesis (stimulating continued root development), pulpectomy, apexification, or revascularisation procedures. Current research has focused on using signal proteins, such as vascular endothelial growth factor (VEGF), which stimulate angiogenesis and vasculogenesis for tertiary dentin formation. Several studies have shown that VEGF enhances pulp cell proliferation and neovascularization, and increased the formation of reparative dentin in dental pulp [11, 12]). Research conducted by Yadlapati et al. [13] showed that VEGF-loaded polymer fibre as a growth factor-loaded scaffold is a viable option for pulp tissue regeneration.

Apexification is considered the treatment of choice for young permanent teeth with necrotic pulp and an open apex but with adequate root length and root–dentine thickness [6]. The most commonly advocated treatment options for apexification are either long-term dressing with calcium hydroxide (CaOH) [14], or placement of a calcium-silicate cement, such as mineral trioxide aggregate (MTA), to create a physical apical barrier. MTA is considered to have advantages over traditional intra-canal medicaments and includes expediency of treatment, the longevity of the apical seals, excellent biocompatibility, and osseo-induction [15, 16]. The exact mechanism of apexification and the quality of the hard tissue formed remains poorly understood.

## 2. Clinical Case

**Figure 1:**
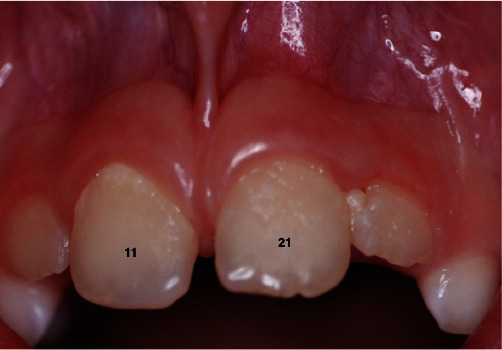
Presentation of teeth #11 and #21 after one month of traumatic injury.

Written informed consent was obtained from the patient for the treatment and publication of this case report. An eight-year-old female Caucasian patient slipped over in the bathroom and suffered a traumatic injury of teeth #11 and #21 with minor enamel infractions (class I) to both teeth due to an intrusive force. Tooth #11 was restored immediately after the injury with a simple resin composite restoration. One month following the traumatic injury, the patient presented with discomfort, infra-occlusion, and swelling in the buccal sulcus over the apex of tooth #21 (Figure 1).

**Figure 2:**
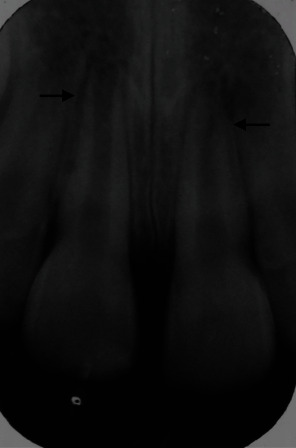
A periapical radiograph one-month following trauma illustrating a wide-open apex on tooth #11, and evidence of resorption on two-thirds of the distal aspect (arrow). There is evidence of spontaneous apexification (arrow) on tooth #21 with a radiolucent area on the distal aspect of the root close to the apical dome.

Upon examination, tooth #21 did not respond to sensibility testing. Radiographic examination revealed a wide-open apex on tooth #11 consistent with the patient's age, with evidence of resorption on the distal aspect of the root (Figure 2). For tooth #21, there was radiographic evidence of spontaneous apexification in addition to a periapical radiolucent area on the distal aspect of the root near the induced apical dome.

**Figure 3:**
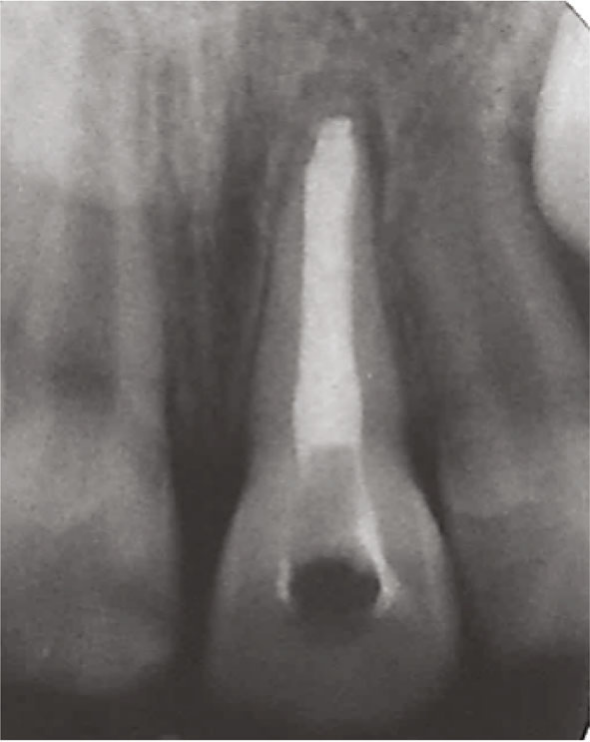
Posteroanterior radiograph after obturating with MTA (6 months after initial root canal treatment).

Following endodontic diagnoses for tooth #21 of necrotic pulp and acute apical abscess, the root canal of tooth #21 was accessed under a dental dam, and following light instrumentation was irrigated with sodium hypochlorite. It was noted that there was an apical barrier with a dimension approximately the size of a No. 30 K-file. The canal was then dressed with CaOH (Dentsply, Yucaipa, CA, USA) and temporarily sealed with Intermediate Restorative Material (IRM) (Dentsply). After one month, the tooth was symptomless, and the canal was redressed with CaOH. The patient returned six months following the initial treatment, and tooth #21 was again noted as being asymptomatic. The canal was accessed under a dental dam, irrigated with sodium hypochlorite, and dried. There was a detectable barrier at a working length of 20 mm, and the entire root was obturated with MTA (Dentsply Sirona, Auckland, New Zealand; Figure 3). The access cavity was restored with a layer of glass ionomer (GC Corporation, Tokyo, Japan) followed by resin composite (3M ESPE, St. Paul, MN, USA).

**Figure 4:**
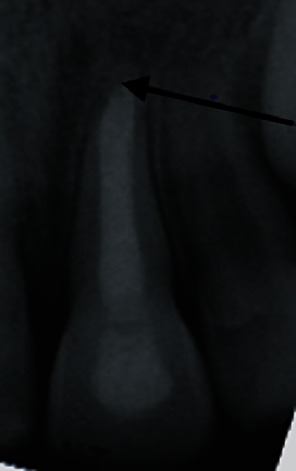
Posteroanterior radiograph of tooth #21 four months after obturating with MTA.

The tooth was reviewed four months after obturation and noted as being slightly tender to percussion, but no other symptoms were noted. A periapical radiograph indicates evidence of periapical healing (Figure 4).

**Figure 5:**
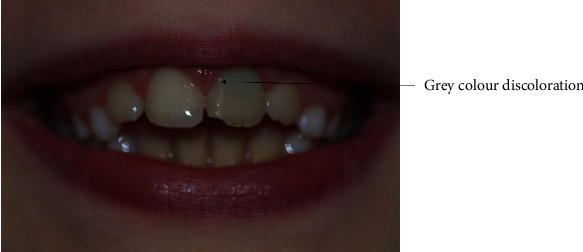
Photograph taken during the review appointment at 19 months after obturation with MTA showing a grey discolouration on tooth #21.

The tooth was then reviewed 19 months after obturation. The tooth was asymptomatic, but the crown had a grey disclouration (Figure 5). After discussion with the patient and parents, the resin composite was removed under a dental dam, and the access cavity was filled with sodium perborate/water, then sealed with IRM. After two weeks, the tooth had whitened satisfactorily, and the access cavity was again sealed with resin composite.

**Figure 6:**
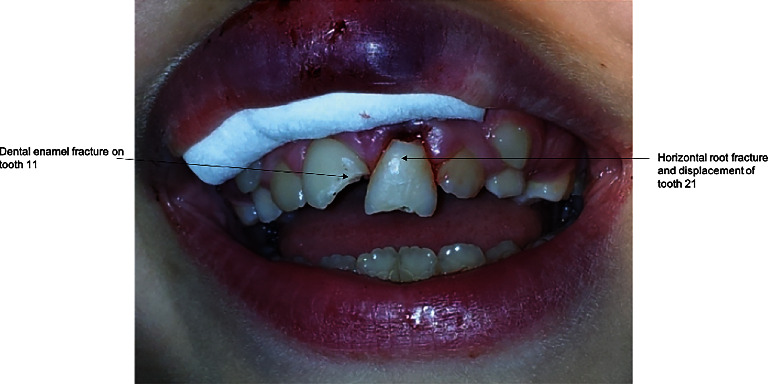
Photograph of the dentine enamel fracture sustained by tooth #11, and the crown root fracture incurred by tooth #21.

Two and half years following the initial injury, tooth #11 sustained a dentine enamel fracture, whereas tooth #21 incurred a horizontal root fracture with the coronal portion being extruded (Figure 6).

**Figure 7:**
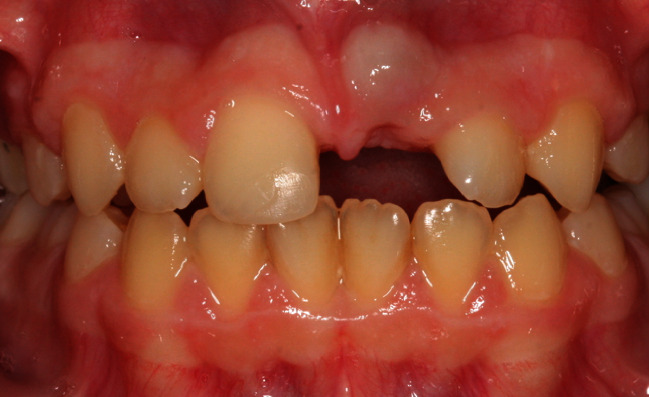
Photograph following decoronation of tooth #21 and root submergence.

Due to the location of the horizontal fracture being approximately 2 mm subgingivally, tooth #21 was decoronated, and the root was submerged (Figure 7).

**Figure 8:**
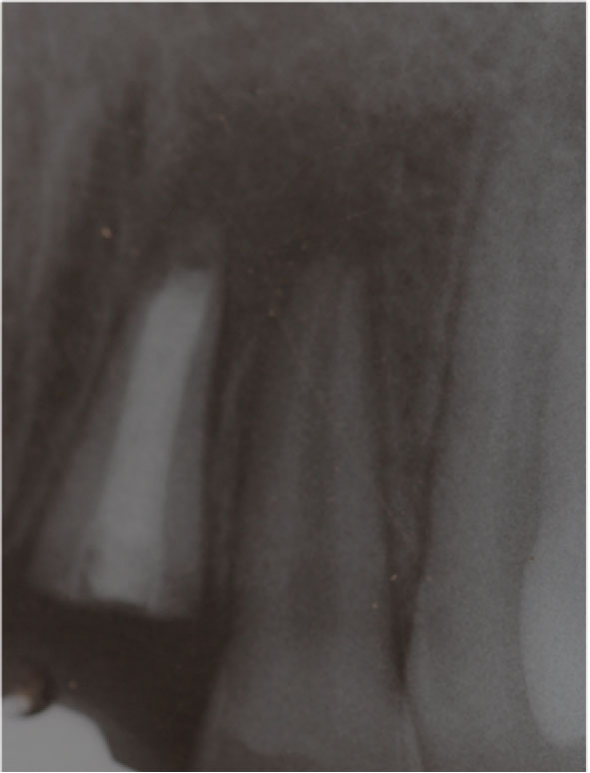
Periapical radiograph revealing large radiolucent area associated with the apex of tooth #21.

Three years following the second traumatic injury (5.5 years after the initial traumatic injury), the patient presented with pain and swelling associated with the root of tooth #21. A periapical radiograph revealed a large radiolucent area associated with the apex of tooth #21 (Figure 8).

**Figure 9:**
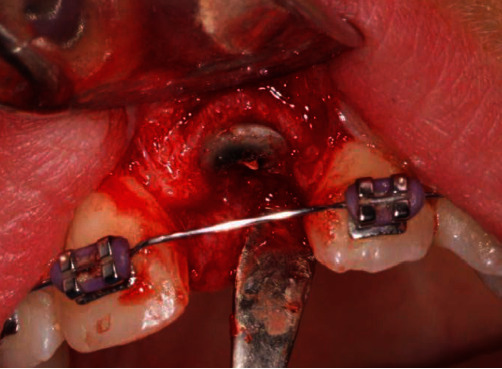
Extraction of the root of tooth #21, and evidence of good marginal bone quality.

**Figure 10:**
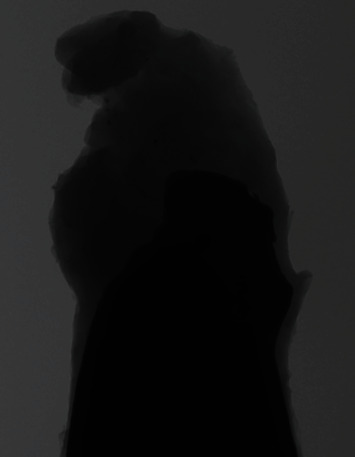
Micro-computerised tomography (CT) radiograph of the soft tissue mass and apex of tooth #21.

The patient consented to the extraction of the tooth root and a ridge preservation procedure (Bio-Oss/Bio-Gide; Geistlich Pharma, Chatswood, NSW, Australia) to maintain the residual bone volume. Excellent bone quality was noted at the marginal aspect of the tooth during extraction (Figure 9), and the site healed uneventfully. A soft tissue mass was found to be attached to the apex of tooth #21 (Figure 10).

The root end and soft tissue mass of tooth #21 were examined using a Skycan 1172 micro-CT analyser (SkyScan, Aartselaar, Belgium). The voltage and the current of the micro-CT scanner were set to 50 kV and 190 *μ*A, respectively, with a focal spot size of <5 *μ*m. The camera was set to a pixel size of 11.45 *μ*m, and the two-dimensional (2D) images were obtained by rotating the sample 180° with a rotation of 0.4° per sector. A total of 2062 2D images of the transverse sections of the root end were recorded with a total acquisition time of 20 minutes.

**Figure 11:**
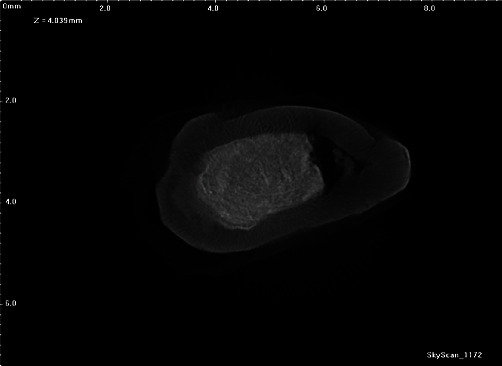
Micro-CT radiograph showing obturation with MTA.

Micro-CT analysis of the root demonstrates evidence of an apical calcification or dome following the initial traumatic injury. The obturation with MTA is demonstrated in Figure 11 with some evidence of hard tissue calcification on the distal aspect.

**Figure 12:**
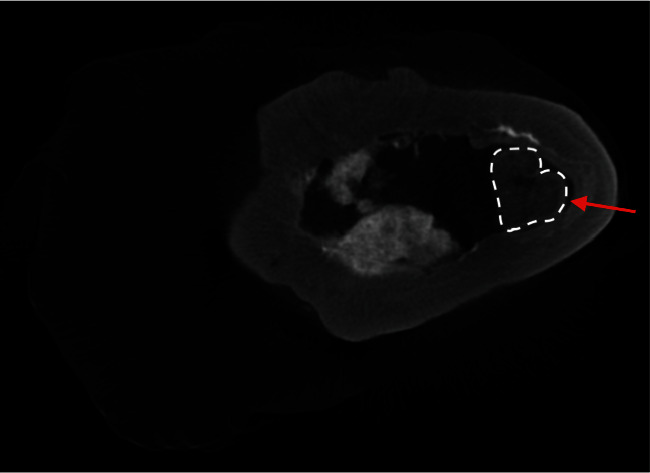
Micro-CT radiograph showing clinical evidence of apical calcification following the injury (red arrow).

Further apically there is further evidence of apical calcification, which can be demonstrated in Figure 12 (shown by arrow). The calcification is irregular and shows a variable increase in radiopacity compared with the surrounding dentine.

**Figure 13:**
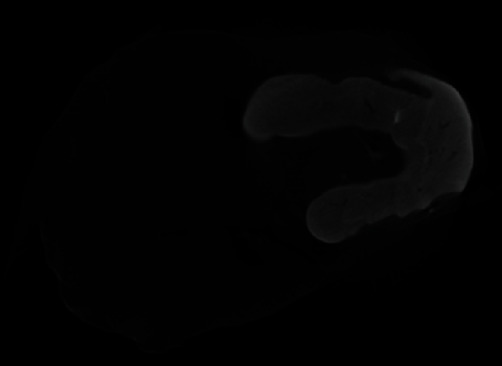
Micro-CT radiograph of the apical region, in which the root face is no longer continuous Slice 3039.

As slices are viewed further apically there is a region, in which the root face is incomplete resulting in a persistent communication between the root canal and the periodontium (Figure 13).

**Figure 14:**
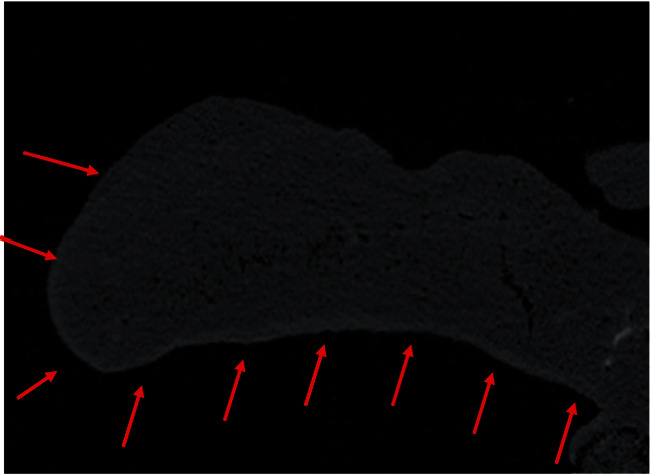
Enlarged image of Figure 13 shows the appearance of cementum-like mineralisation.

**Figure 15:**
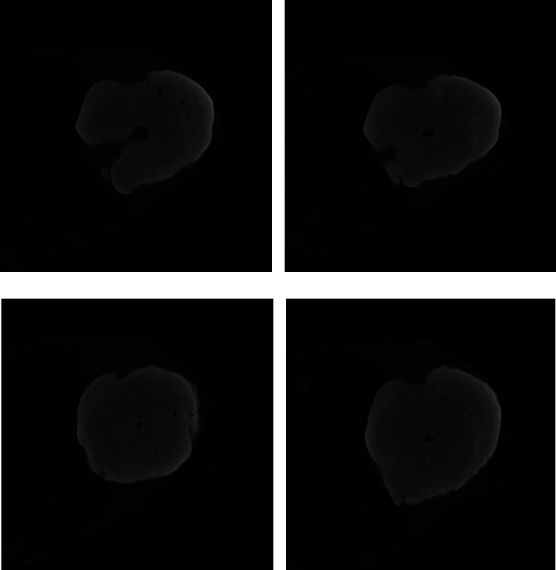
Micro-CT radiograph illustrating the continuous calcification of the root with the formation of a dome with a central lumen lined by minerals consistent with the appearance of cementum. (a) Beginning of calcification. (b) Continous calcification. (c) Formation of the apical dome with numerous channels. (d) Final formation of a calcified Dome.

As the slices are viewed further apically, continuous calcification has occurred (Figure 14) with the formation of a “dome” of calcified tissue (Figure 15).

**Figure 16:**
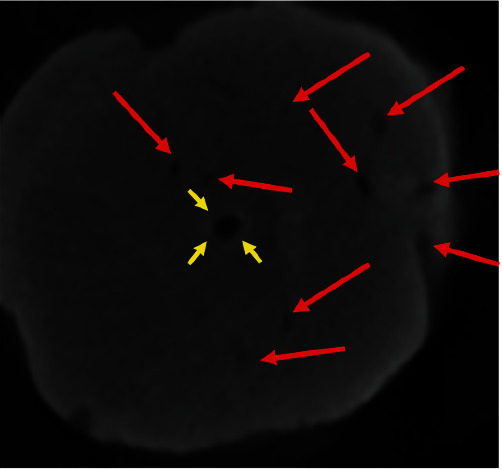
Enlarged view of Figure 15(c). Red arrows indicate some of the numerous channels present in the apical dome.

An enlarged view of Figure 15(c) has been highlighted in Figure 16 with red arrows indicating some of the numerous channels present in the apical dome. The probable reason for the appearance of the centrally placed “lumen” indicated by yellow arrows, is that it is lined by cementum laid down on the surface of the defect.

**Figure 17:**
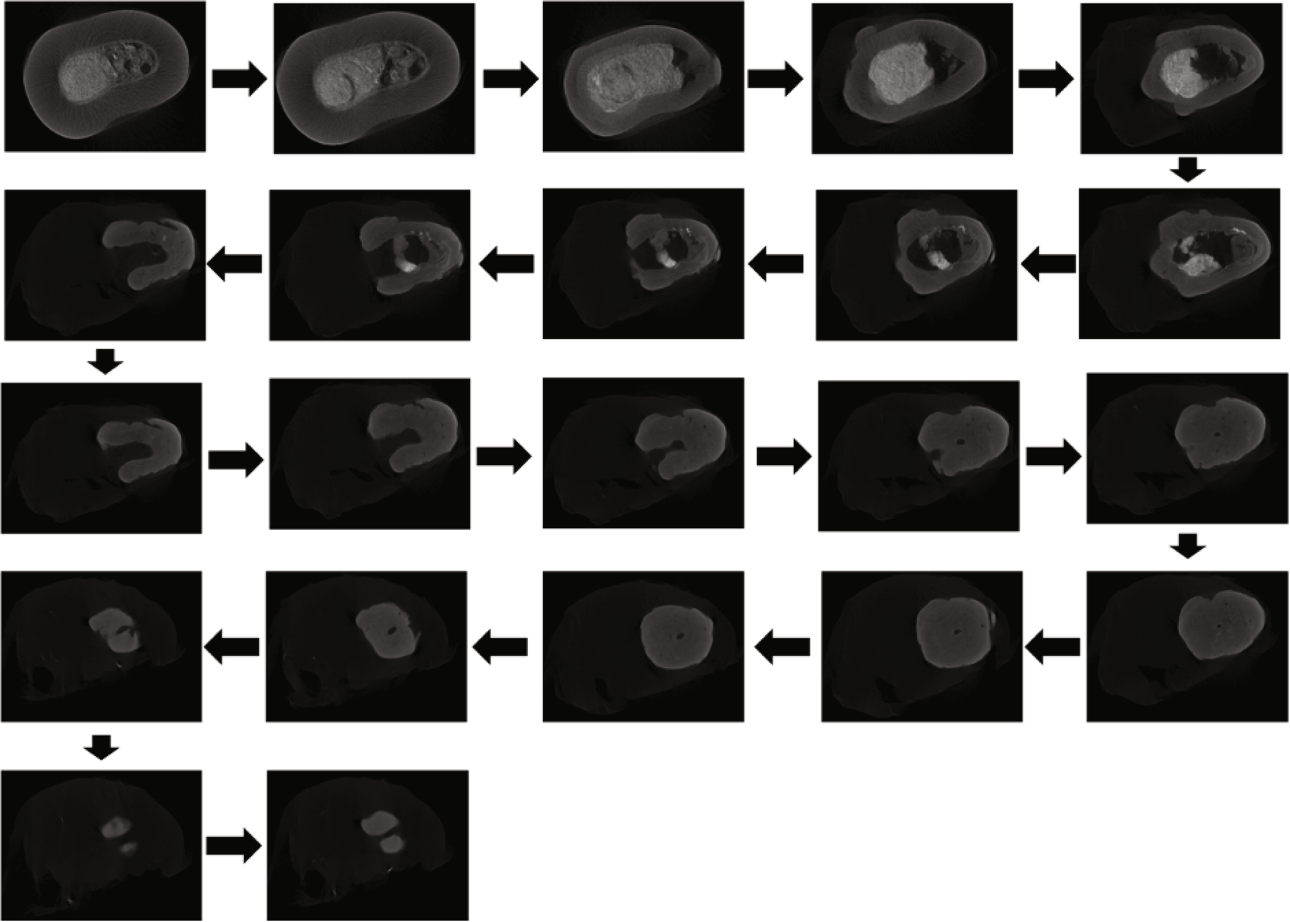
Selected serial sections of micro-CT images demonstrating the development of the apical calcification.

Viewing selected serial selections of the micro-CT images as a whole indicates the total healing response and development of the apical calcification (Figure 17).

## 3. Discussion

The use of micro-CT on a tooth that has undergone two separate traumatic events provides insight into the healing potential of immature teeth and the apexification process observed in limited cases of spontaneous apexification. “Spontaneous apexification” is defined as the rare occurrence of continuous growth and development of an apical calcific barrier after trauma or pulpal necrosis without the external intervention of endodontic procedures [6].

The impact of the teeth on the bathroom floor caused an intrusive injury as well as an enamel fracture. One month after the traumatic injury, there was radiographic evidence of apical calcification without any clinical intervention on tooth #21 compared with tooth #11 (Figure 2), and the apical closure was confirmed clinically as a No. 30 hand file was binding in the apical portion. The slice taken just apical to the subsequent MTA placement (Figure 11) would be consistent with a 0.30 mm apical diameter. Nineteen months after obturation with MTA, tooth #21 showed a grey discolouration. The discolouration would have occurred due to the presence of the radio-opacifier, bismuth oxide present in calcium silicate cement, such as MTA [17, 18]. During the time of the treatment, root canal sealers, such as Endosequence and AH Plus, were not available in the market, which maintains colour stability over a long period [19].

The calcification is not regular as would be expected by normal dentine deposition by odontoblasts and there is evidence of a lateral opening or communication with the periradicular tissues, which is consistent with the observations described by Heithersay [14].

There are a limited number of case reports describing this rare finding where apexification occurred without the benefit of endodontic treatment, and the term “auto-apexification” or “spontaneous apexification” has been described by Barker and Mayne [20] and Kahler and Heithersay [6], respectively. The time frame of 1 month following injury to have clinical evidence of apical calcification is short. A limitation of this case report is that there were no radiographs before the patient presented with pain to prove that an unrelated traumatic event may have occurred historically. However, when the parents were further questioned on this, there was no recollection of a significant additional traumatic injury.

Following the initial trauma, the pulp of tooth #21 would have been in a compromised state, which ultimately resulted in pulp necrosis, most likely within 1 month of the injury. In this case, apical calcification occurred without clinical intervention, such as pulpotomy, pulpectomy, or revascularisation/regeneration techniques (Huang et al., 2020). Vojinović and Vojinović [7] provide evidence that the retromigration of cells from the periodontium into the apical papilla stump is possible under pathological conditions and that together with other apical pulp cells of immature teeth, may create a calcified tissue resulting in apexification. Normal root development depends on the survival of stem cells of the apical papilla, the presence of epithelial cells derived from HERS, or its defragmentation as epithelial rests of Malassez ([21]). Palma et al. [22] undertook a histologic evaluation of infected immature dog teeth that had received either regenerative endodontic procedures or apexification with MTA and reported that homogenous mineralized tissues were found nearby and in continuity with the cementum bridges of the apical barriers. The authors suggest that the mineralised tissue consisted of the apical papilla and that it had interacted with the HERS allowing for differentiation to take place. It is, therefore, possible that the mineralisation of stem cells of the apical papilla occurred in this current report.

Kahler and Heithersay [6] reported a case of spontaneous apexification, in which the pulp was necrotic, and the tooth had periradicular pathosis. A seven-year-old child sustained a bicycle accident and after six months there was evidence of an apical radio opacity. No treatment was provided, and nine years later, when the patient presented with a labial swelling a radiograph revealed a “large root canal space in coronal three-fourth of the root, capped by an apical dome. A radiolucency was evident adjacent to the junction of the wide coronal segment and the apical dome.” The radiolucency observed by Kahler and Heithersay [6] could be consistent with the incomplete root appearance seen in this case report (Figure 13).

As more apical scans are viewed, representative of a time course of calcified tissue development, the incomplete root becomes a continuous root end with a patent canal. This is consistent with the observations of Heithersay in the management of pulpless incompletely developed teeth that are managed with the application of CaOH [14].

The management of the initial trauma on tooth #21 included restoring the access cavity with a layer of glass ionomer (GC Corporation) followed by resin composite (3M ESPE). A recent case series report by Santos et al. [23] demonstrates that a corono-radicular adhesive restoration contributes to good long-term outcomes on teeth with open apices that have undergone apexification procedures. It is possible, that if the restoration in the current case extended further into the radicular portion, then the tooth may have been more resistant to fracture.

As a result of the second traumatic injury, there was a horizontal fracture of approximately 2 mm subgingivally. It was determined that the tooth was non-restorable, and the tooth was decoronated with the root submerged to help maintain alveolar height and width during continued growth for the placement of an implant in the future. Unfortunately, the patient suffered pain and swelling associated with the submerged root, which necessitated the extraction of the root. A ridge preservation procedure using Bio-Oss/Bio-Gide was undertaken to maintain the residual bone volume. An alternative technique to Bio-Oss is the use leukocyte–platelet-rich fibrin membranes and plugs [23, 24].

Comparing the original radiographs after the initial placement of MTA (Figure 3) and the follow-up before extraction (Figure 8), it is clear that there has been a loss of MTA from the canal in the apical and coronal portions. From the micro-CT, it appears as though there is a root fracture (Figure 12), which could account for the breakdown of the MTA filling on the distal aspect and indicates that the material may be soluble over time if there is a fracture and/or concurrent infection.

The calcific barrier that was established six months following trauma revealed radiographic and clinical evidence of successful apexification. The MTA root filling was well adapted to the root end, and there was the appearance of intact lamina dura. Only with the ultimate demise of this root following a second injury has it been possible to examine the root end carefully. The use of micro-CT demonstrated that the apexification was incomplete and is a reminder that periapical radiographs provide only a 2D view and may at times be misleading. Apexification should be regarded as complete, or incomplete, and with the development of cone-beam CT, the determination of this may become more predictable. It is interesting to note that the apical dome of calcified tissue had a central canal. It is unlikely that there was any vital pulp tissue in this apical portion and represents another example of the complex interplay between periradicular tissues with cells that are not fully differentiated.

## 4. Conclusions

Due to a rather unique set of circumstances of a traumatic injury on an immature tooth, followed by a subsequent second trauma, which ultimately resulted in extraction, the presented case report highlights a rare case of spontaneous apexification using radiographic and micro-CT analyses. The calcification was irregular, and slices apical to the level of obturation with MTA demonstrated an area of the root face being discontinuous, with the root canal being in direct communication with the periodontal ligament. Slices further apical demonstrated that the canal became continuous again, with a central lumen. Micro-CT has a high degree of resolution and has provided further insights into the apexification process without destruction of the tooth, which occurs with normal histological processing.

## Data Availability

Data supporting this research article are available from the corresponding author or first author upon reasonable request.
